# Understanding Tuberculosis: Perspectives and Experiences of the People of Sabah, East Malaysia

**DOI:** 10.3329/jhpn.v28i2.4880

**Published:** 2010-04

**Authors:** Christina Rundi

**Affiliations:** Public Health Division, Sabah Health Department, Ministry of Health, Malaysia

**Keywords:** Knowledge, attitudes, and practices, Qualitative studies, Perceptions, Tuberculosis, Malaysia

## Abstract

Malaysia is a country with the intermediate burden of tuberculosis (TB). TB is still a public-health problem in Sabah, one of the two states in East Malaysia. In 2007, the state of Sabah contributed slightly more than 3,000 of 16,129 new and relapse cases reported in the country. It has a notification rate of two and a half times that of the country's. Very few studies on TB have been conducted in Sabah, and there is little documentation on the perceptions of TB patients and the community about TB, healthcare-seeking behaviour, and impact of TB on the people of Sabah. A qualitative study was conducted in 2006 in seven districts in Sabah to assess the knowledge and perceptions of TB patients and the community about TB, also to know the experiences of healthcare services, and to examine the impact of TB on patients and families. Purposive sampling identified 27 TB patients and 20 relatives and community members who were interviewed using a set of questions on knowledge, perceptions about TB, healthcare-seeking behaviour, and impact of TB. A further 11 health staff attended informal discussions and feedback sessions. Most interviews were taped and later translated. Data were analyzed using thematic content analysis. Ninety-six percent of the respondents did not know the cause of TB. Some thought that TB occurred due to a ‘tear’ in the body or due to hard work or inflammation while others thought that it occurred due to eating contaminated food or due to sharing utensils or breathing space with TB patients. Although the germ theory was not well-known, 98% of the respondents believed that TB was infectious. Some patients did not perceive the symptoms they had as those of TB. The prevailing practice among the respondents was to seek modern medicine for cure. Other forms of treatment, such as traditional medicine, were sought if modern medicine failed to cure the disease. TB was still a stigmatizing disease, and the expression of this was in both perceived and enacted ways. TB also affected the patients in various aspects of their lives, such as psychosocial, physical, financial and life practices. Patients who were farmers complained that they did not recover fully from their disease and were not, thus, able to continue with their previous work. Patients changed their life practices, such as not sharing their utensils, had a separate sleeping area, and practised social distancing. On the other hand, most health workers were unaware of the effects of TB on their patients and that knowledge of their patients on TB was inadequate. There is a need to understand the reasons for the misconceptions about TB and to address the lack of knowledge on TB through health education. Patients need to recognize the symptoms of TB early so that prompt treatment can be initiated, and patients need to be convinced of its curability.

## INTRODUCTION

Tuberculosis (TB), a disease of ancient time as revealed by DNA analysis of tissue samples from mummified bodies and skeletal remains of more than 5,000 years (1), is still a public-health problem. It is one of the most important yet neglected international health priorities (2). In 2007, it was responsible for an estimated 1.32 million deaths among HIV-negative people and an additional 456,000 deaths among HIV-positive people (3). Based on surveillance and survey data, the World Health Organization (WHO) estimated that 9.27 million new cases of TB occurred in 2007, a rise of 30,000 from the previous year (3). The sufferings of TB patients, in terms of physical and economic consideration, have been reported, including rejection as a result of the stigma associated with TB (4–10).

TB is endemic in Malaysia with a notification rate among smear-positive patients of 36 per 100,000 in 2007 (11). Sabah contributes one-third of the total cases in the country and has a notification rate for all cases of 100–200 per 100,000 people for almost a decade now (12). Sabah, one of the 13 states in Malaysia, is located in the Borneo Island. The major ethnic groups are: Kadazandusuns, Muruts, Bajaus, and Rungus. Although the majority profess to either Islam or Christianity, some still hold on to ancient beliefs and practices.

TB is a social disease, and healthcare-seeking behaviour among patients is influenced by gender, age, socioeconomic and social status of female, type of illness, access to services, and perceived quality of the service (13), which often interact in a complex web. People can be confused as to the implicationss of TB symptoms, costs of transportation, the social stigma, the high cost of medication, and perceptions of patients about clinic facilities as unfriendly, and all these contribute to the complexity of the disease (4). To tackle the huge problem of TB in Sabah, it is important to address all these issues. Very little is known about healthcare-seeking behaviour of the people in Sabah with regard to TB. The specific objectives of the study were to assess the knowledge and perceptions of TB patients and the community about TB and the experiences of healthcare services and to examine the impact of TB on patients and their families. The findings presented here are based on a qualitative study, which explored healthcare-seeking behaviour with regard to TB among the people of Sabah and the impact of TB on patients and their families.

## MATERIALS AND METHODS

### Study population

Thirty-two indigenous groups comprise the people of Sabah, with over 80 locally-spoken dialects with a wide variation in traditions and cultures. This study was conducted in seven districts: Kota Kinabalu, Penampang, Putatan, Tuaran, Kota Marudu, Kudat, and Keningau. Kota Kinabalu is the state capital with a mixture of all ethnic groups. Penampang is a predominantly Kadazan area where one-third of the population belong to this ethnic group while the neighbouring district of Putatan is a predominantly Malay area (57%) (14). Around 45% of the people in Tuaran are Dusuns, followed by Bajaus (29%). Similarly, around half of the people in Kota Marudu are Dusuns. In contrast, two-thirds of the population of Kudat are Rungus while in the interior district of Keningau, two-thirds are of the Kadazan-Dusun-Murut ethnic groups (14).

The author developed a conceptual framework to facilitate the exploration of healthcare-seeking behaviour among the respondents. In this framework (Fig.), the author has combined the elements from the Health Belief Model (15), Health Care Utilization Model (16), the four ‘As’ (Fig.) and the pathway model (17). The perceptions of patients on severity and benefits to therapy-choices help conceptualize healthcare-seeking behaviour. These perceptions are influenced by other factors, such as psychological, socioeconomic, and gender. The roles of ‘significant others’ and need and enabling factors also provide cues for taking action for treatment. The enabling factors include availability, accessibility, affordability, and acceptability of therapy-choices. Based on this framework, the main areas explored were recognition of symptoms, healthcare-seeking behaviour, perceptions about illness, perceptions about care, gender difference in seeking healthcare, effects of TB on patients and family members, and reactions of the community towards those with the disease.

**Fig.**
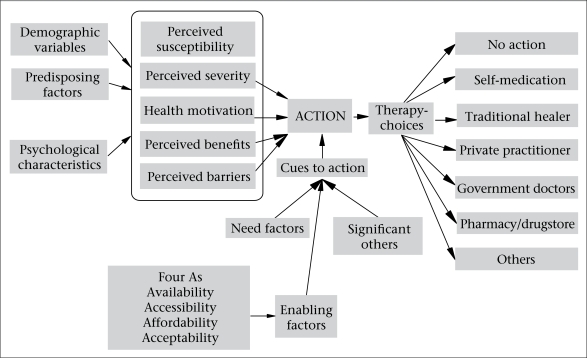
Conceptual framework for health and health seeking behaviour among tuberculosis patients

### Sampling methods

Purposive sampling was used for looking for information-rich respondents, and a sampling matrix was created to identify them (Table 1). The health staff of the selected districts assisted in identifying respondents from each group.

**Table 1.** Sampling matrix for identifying respondents

Group	Income level and location	Gender	
		Male	Female
	Low income		
TB patients	Rural	XXXX	XXXX
	Urban	XXXX	XXXX
	High income	X	X
Traditional healer		X	X
Family member or relatives or other key informants	Rural	XX	XX
	Urban	XX	XX
Health staff		XXXX	XXXX

### Conceptual variables

Data collected for the conceptual variables during in-depth interviews were on local terminologies of TB, recognition of symptoms, perception of TB patients and the community about illness, healthcare-seeking behaviour, knowledge on TB, perceptions about care, effect of disease on the patient and family, and the community perceptions about TB. Health staff were asked questions on reasons for seeking treatment by patients and knowledge of patients on TB.

### Collection of data

In-depth interviews were performed on 27 TB patients from seven districts and on 20 other respondents who were either spouses, relatives, or other people in the same village as the patients. Interviews were carried out as agreed by the respondents in terms of time and place. Seven respondents were interviewed in the nearest clinics and the remaining respondents in the home. Translators were needed for five interviews. The choice of translator was important, and to ensure confidentiality, they were not recruited from the same village as the respondent. Only two translators were needed throughout the study. All interviews, except for the first two, were tape-recorded with verbal permission from the participants. The health staff participated in the discussions and feedback sessions in the clinics during visits to selected districts. These were informal sessions to gather information, such as on reasons for seeking healthcare.

### Analysis of data

All in-depth interviews took 60–90 minutes. Transcription of interviews was performed immediately and in the language in which the interview was conducted. Each transcription took 4–6 hours. These were then translated to *Bahasa Malaysia* (national language). Analysis of data was done in several stages throughout the study in the national language, using thematic content analysis. The first stage was to find common themes, such as symptoms, causes, healthcare-seeking behaviour, and perceptions about care. The second stage was a more rigorous process of analyzing the content for the emergence of new categories and sub-cate-gories. This was particularly important in identifying the various categories of the effects of TB and stigma. The third stage involved synthesizing the findings to look for relationship between the various themes to create a clearer understanding of TB as seen from the patients and the community perspective.

Collection and analysis of data were performed by the author.

Ethical issues, such as autonomy, confidentiality, and anonymity, were observed throughout the study. The ethical approval for the study was obtained from the Ministry of Health, Malaysia.

## RESULTS

### Characteristics of participants

In total, 27 patients and 20 non-patients, such as spouses, relatives, villagers, and traditional healers, were interviewed in the seven districts. Eleven members of health staff were involved in the discussion and feedback sessions. Table 2 shows the characteristics of all respondents.

**Table 2.** Characteristics of respondents

Variable	TB patients (n=27)	Non-TB patients (n=20)	Health staff (n=11)
Age (years)			
<20	1	0	0
20–29	3	1	4
30–39	7	4	3
40–49	5	6	4
50 and above	11	9	0
Gender			
Male	15	7	5
Female	12	13	6
Ethnic group			
Kadazan	4	1	1
Dusun	6	2	3
Murut	3	2	1
Rungus	7	10	2
Bajau	4	4	2
Others*	3	1	2
Religion			
Islam	10	8	7
Christian	15	9	4
None/others	2	3	0
Occupation			
Farmer	7	2	
Housewife	6	7	
Retiree	2	0	
Self-employed	4	3	
Unemployed	4	1
Traditional healer		2	
Village headman	0	1
Others**	4	4

*Brunei, Sungai, Chinese, and Indian;

**Clerk and fisherman;

TB=Tuberculosis

### The community perspective of TB

TB was relatively unknown in Sabah in the 1960s and was often described by its symptoms (chronic cough, coughing out blood or being very thin) as *batuk kering* (dry cough) or *mengurus* (becoming thin). TB was not regarded as ‘the works of the evil spirit’, and, thus, the role of spiritualist was minimized. The two traditional healers who were in their 50s said that they were not taught on how to treat TB. According to them, such a disease did not exist when they started their training at the tender age of 10 years. Instead, they believed that TB was due to hard labour and contaminated food and could be treated through traditional rituals, such as sacrificing pigs. Traditional medicinal plants were also used by the local inhabitants for generations to relieve symptoms of many diseases and cure various infections. This knowledge was important for their survival, especially among those who live in the interior. The two traditional healers had also used medicinal plants for treating their patients with chronic cough and loss of weight.

When asked as to the cause of TB, the prevailing notion was that there was a ‘tear’ inside the body which eventually became ‘rotten’ making it possible for ‘some thing’ to enter the ‘tear’ and caused TB. The ‘tear’ could be due to several reasons; the main among these was hard work or inflammation of the stomach-lining (gastritis) (Box 1).

**Box 1**

“It is due to hard work, so something inside got torn due to hard work. The torn area then becomes rotten, that is what people say.” (A 66-year old farmer)

“According to the people here, the origin of TB, number one is gastritis because he did not look after his food (nutrition). You know how it is with this village people …. sometime when they go to work … they have not eaten, not taken any drinks. Hah, that is the cause.” (A 27-year old fisherman)

Some patients believed that they got the disease due to work and exposure to rain and sweat. Others believed that TB was caused by eating or drinking contaminated food; food that have been exposed to dirt, dust, or even the saliva of TB patients. There were some who agreed that TB was caused by germs but according to them, that did not explain how one got the disease. One patient thought that TB was hereditary because all his family members had TB, except for him and his sister. He was not surprised when he was told that he had TB. A 25-year-old clerk believed that she was weakened by TB, which made it easier for her to be ‘charmed’ (Box 2).

**Box 2**

In May, I felt this sharp pain just below my ribs. It felt painful when I cough, like being sliced by a knife. My uncle took me to see a *bomoh* (traditional healer). You see, last year, someone cast a charm on me. I had to be treated by four different *bomohs*. Before I went to see the traditional healer, I went to a private doctor and asked for a scan over the area. I told my family and the *bomoh* that I am on treatment for TB but they told me to get treatment anyway because I may have two illnesses. (When asked whether she thought that she had TB because someone had ‘charmed’ her, she had this to say). No, but maybe because I am already weakened and sick by TB, then it was easier for him to cast the ‘charm’ on me.

Another TB patient believed that a ‘charm’ has masked his disease, which was not detected despite repeated sputum and chest X-ray examinations. After his mother-in-law had sought the help of an experienced healer, a visit to the clinic revealed a positive sputum examination, and the X-ray revealed damage to the right side of his lungs.

Ninety-eight percent of the respondents agreed that TB was infectious and can spread through sharing of eating utensils and through face-to-face interaction. Among some cultures in Sabah, many social gatherings involved drinking local rice wine often served in a jar and sipped through bamboo straws. Many people of this culture opined that sharing cups and plates and also sharing the bamboo straws were routes for the transmission of TB. Due to this belief, TB patients did not share utensils or meals with family members and others.

### Healthcare-seeking behaviour

Cough was the commonest symptom, followed by loss of weight. The other respiratory symptoms experienced were haemoptysis (coughing out blood), shortness of breath, and chest pain. Loss of weight was the commonest constitutional symptom, followed by fever. Other constitutional symptoms included loss of appetite, difficulty in sleeping, and lethargy. Some patients knew that they had TB because they have heard of it or have experienced it in their family. Other patients thought that they were suffering from other diseases but had no idea about what these were. Others thought that they had diseases, such as diabetes, asthma, gastritis, breast cancer, and common cold. Among the non-patient respondents, cough and/or coughing out blood and loss of weight were identifiable with TB. The large majority (74%) of the patients sought treatment due to worsening of symptoms. If cough did not get worse, such as by becoming productive and persistent, this symptom would be ignored for a long time. However, if cough was accompanied with blood or difficulty in breathing or lethargy, treatment was sought promptly.

In most instances, patients rely on modern medicines for cure. Other options were also considered when modern medicines failed to cure the disease completely. One patient had sought treatment from a traditional healer before getting treatment from a government clinic because her family thought that she had been poisoned (Box 3).

**Box 3**

“Yes … for about a month, she drank the traditional medicine from the *bomoh.* After that … when there was no improvement, my father-in-law said … let us bring her to the hospital. First time we went, they could not find what was wrong with her, they just examined her and said that she had common cough. My father-in-law was not happy. Let us bring her back to the hospital and ask for an X-ray. That is why the second time I took her, she had an X-ray done, and then we were told that she has TB.” (A patient's husband)

Some people may not admit that they sought treatment from traditional healers as this was considered a pagan practice. Most (81%) patients decided on their own as to when and where to seek treatment, although this was often influenced by family members and relatives. In the past, village headmen were consulted regarding treatment of diseases but such was no longer the case.

### Impact of TB

All the TB patients in the study eventually completed their treatment. However, many, particularly men, felt that, as a result of TB, they often felt weak and never fully recover to their pre-illness physical state. Patients who were farmers find it difficult to continue farming due to residual weakness (Box 4).

**Box 4**

“Of course, it is difficult. Before I had this disease, I was only old. But whatever I want to do, I did it on my own. But now that I have this disease, although I am almost cured, according to the doctor, and my own feelings, I will definitely not recover completely as before. So, definitely it is difficult because before this illness, whatever work, I did it myself. But now, even near the house, even when I see all the plants withered away, I cannot do anything.” (A 60-year old farmer)

Ninety-six percent of the patients told their family members about their disease, and some also informed church members, their superiors, and close colleagues at workplace. The reasons for not telling other people outside the family-circle included worry over their reaction. Some felt that others did not need to be told and considered it embarrassing to be infected with TB. One respondent was worried that people might equate TB with AIDS and, thus, distanced themselves from him.

All the patients initiated changes in their everyday living as a result of TB. These include separation of utensils, new sleeping arrangement, and reduced social contacts and activities. New sleeping arrangement could cause problems between husband and wife as revealed by a 45-year old housewife (Box 5).

**Box 5**

“When my husband had TB, if I am not mistaken, I did not sleep with him for seven months. He had to sleep on the floor while I slept with our second child in the bed. I think, for the first three months, he was hurt by that but I told him that it is not that I am neglecting his needs but this is for our own good.”

In two rather extreme cases, the patients moved out of the house temporarily during the treatment period against the wishes of their families. One patient rented another flat not far from his family during his treatment period for fear of infecting his baby son. Another patient moved to a small barn which also served as a store for rice-stock for the family. Instead of being left on his own, his children, grandchildren, and relatives would sit and eat together with him and play in the barn to give him company. One patient was asked by his family to live in their farm-house and looked after their farm. He sold the farm-produce for money, and when there was none to sell, he begged for money from friends and the public.

Due to the stigma of the disease, TB patients who are single faced a poor prospect of marriage. This was revealed in an interview with a village headman who was not a TB patient. In the local context, a village headman has considerable influence on the cultural aspects of the community (Box 6).

**Box 6**

When he comes to our house, as usual, during meal-time, we feed him too. After that, we separate his bowl and plate. After he has left, we wash his bowl and plate, first with soap-water, then with hot water. That is what we have been told. According to our custom, he cannot get married because he is considered sort of physically deformed. After all, he cannot look for job. It means that he can no longer be responsible for his family.

A couple with TB, who operated a small grocery stall in the village, had experienced stigma from the local community. As a result, they had to travel to another clinic, rather than the one in their village, to get their medicine, and in the early stage of their disease, their business was also affected as the villagers did not frequent their stall (Box 7).

**Box 7**

The villagers did not come and buy from our stall for about a month because they were scared. They were scared until I told them, although we are sick like this, we have never brought our plates and beg for food from you. I maybe sick but I can feed myself, I told them. They call us the ‘TB grocer’ and that hurts me. If someone had said that I have TB, I am not ashamed, I did not ask for this. And I am not the only one with TB here.

It seemed that stigma did not disappear after cure. An elderly patient blamed a relative, who had TB, for her disease. Although that relative had completed treatment, she was not sure whether he was completely cured. Stigma (perceived and enacted) might be deeply rooted within all the communities but there are some evidence that stigma might have lessened over the years as observed by a patient (Box 8).

**Box 8**

“This disease is like a disgrace. Last time (in 1987), when my husband was sick, we went to the hospital. When they knew that my husband has TB, they were afraid to come near my husband. They were hospital staff … nurses. Maybe now, this disease is like normal, not like before, too embarrassing to tell others.” (A 45-year old housewife)

### Perception of TB care

Perceptions of the patients and relatives about care provided by the government and private clinics were generally fair. Most (91%) respondents stated that the care was good or that they did not encounter any problems during their consultations with doctors. However, two did not wish to comment, and another two were frustrated with the doctors who could not diagnose their disease early.

The patients received good support from their families in various forms. Some family members supervised their medications, accompanied them for treatment, or volunteered to get their medicines for them. Some even took over the responsibilities of patients, such as tending the farm and cooking for the family. Despite such support, some patients often felt that they were not able to discuss aspects of their disease openly. They were often pre-occupied with what others thought of them, and this pre-occupation affected them emotionally and psychologically. Women tend to be more affected emotionally while men tend to worry over financial and physical aspects. The majority (67%) male patients (n=15) found it difficult to work as they need to go to the clinic regularly for treatment and, therefore, became dependent on others. Sometimes, they had to borrow money from relatives and friends for bus-fares. Other times, they defaulted as they have to earn the money for the bus-fares. Their spouses also felt the same burden (Box 9).

**Box 9**

“This time, it (the medication) was for two weeks. He gave me medicines for every two weeks. Earlier on, he asked me to come every day but I told him, I am a poor man; I do not have my own transport. Everyday I have to spend RM 12 (about US$ 3) for transportation, how can I bear this financial burden, I told the doctor. We discussed for quite some time, and he agreed to supply medicines for two weeks. I said, if for every two weeks, I can afford to bring my wife here (for treatment) but if every day, I said, how can I go out to sea (to fish)?”

TB patients had little support from others, except support from their family members. In Sabah, a non-governmental organization—Sabah Anti-Tuberculosis Association (SABATA)—renders financial assistance and reimburses travelling costs. This needs to be done through the assistance of health staff who are responsible for the treatment of TB patients in their respective clinics.

### Views of health staff about TB patients

Many health staff did not realize the impact of TB on the lives of their patients as there was limited discussion between the health staff and the patients regarding these impacts. The perceptions of the patients and the community about TB were quite different from those of the healthcare providers. The health staff thought that over 95% of the patients understood the germ theory which was often mentioned during the health-education sessions. They did not realize that the knowledge of the patients and the community was poor and that the effects of the disease were considerable. Some were not aware that the patients were embarrassed at being diagnosed or that negative attitudes of health staff may deter patients from seeking treatment early. It is not clear from this study the extent to which the health staff contributed to the perpetuation of stigma. During one visit to a clinic, one health staff was overheard advising a patient to use separate utensil. Whether such advice was given by other health staff could not be ascertained.

## DISCUSSION

It was apparent from the study that the germ theory was not well-known. The large majority (75%) of the respondents associated TB with a ‘tear’ inside the body or due to hard work, eating contaminated food, or exposure to extreme conditions. Some patients still believed in ‘charms’ which made them vulnerable to TB. Most (89%) respondents believed that TB spread through means in which droplets or saliva or breathing space was shared, and one respondent thought that TB was hereditary.

There is a similarity in the belief that hard work causes TB between the Sabahans and other communities, such as the Vietnamese (18), Mexican (4), Achenese (19), and Filippino (20). Ninety-eight percent of the respondents believed that TB was infectious despite the poor understanding about its cause. This could be due to telling them the infectivity of TB by health staff and the need to isolate TB patients in special wards. In the past, TB patients were warded for the entire treatment period which could be as short as six months or as long as a year. However, this practice has changed. In some hospitals in Sabah, TB patients were warded during the intensive phase of treatment (2 months) or if they have none to look after them or when they could not comply with treatment for whatever reason. Such belief on the infectiousness of TB is in contrast to the belief among Vietnamese: if TB was due to hard work (*lao luc*), it is considered not contagious (18).

Most (81%) patients did not attribute their symptoms to TB. This is not surprising as the symptoms are not specific to TB and can wax and wane. This finding is not different from the study done in West Malaysia in which only 1.5% of the respondents attributed their symptoms to TB (21). As TB was not due to the evil spirit, the natural course of action was to seek modern medicines. Even the few patients who sought treatment from the traditional healers later sought modern medicines when they did not get better, perhaps after re-interpreting their symptoms. Traditional medicinal plants have been used by the traditional healers who were interviewed to treat chronic cough. The stem barks of *Caesalpinia sappan of* the Leguminosae family have been known to be used for TB treatment by the local inhabitants (22).

Some reasons for the inadequate knowledge on TB among TB patients could be due to the unavailability of information and insufficient publicity surrounding TB. Another reason could be due to illiteracy in Sabah which is still high. In 2000, 21% of the population aged six years and above never attended schools, and these ranged from 7% among the Chinese to 21% among the Bajaus (23). As most health-education materials are in the form of pamphlets and posters, the messages could not be accessed by those who are illiterate. The use of other channels of communication, such as television and radio, might be just as ineffective. The electricity coverage for households, especially in rural Sabah, was reported to be 65% before 2004 (24). Proper counselling sessions to TB patients were often not offered by the healthcare provider due to lack of resources and training.

Knowledge on TB which includes the correct understanding of how it can be cured was found to be an important contributor to the completion of treatment (25, 26). However, the extent to which knowledge on TB contributed to the completion of treatment was not explored in this study. Subsequent follow-up on all the TB patients who were interviewed revealed that they had completed their treatment.

Being diagnosed with TB can create the fear of isolation and discrimination. There was evidence of perceived and enacted stigma towards TB patients as expressed through certain acts, such as the use of separate utensils and reduced social contacts. Results of studies in other countries also revealed similar patterns, such as the use of separate utensils among TB patients in Amazon Peruvian community (27), Zambia (10), Kenya (28), and Thailand (29). This could have subsequently contributed to the psychosocial effects on TB patients which resulted in low self-esteem and withdrawal from society, thereby creating a vicious cycle. Studies have shown that social marginalization may influence adherence of patients to treatment (26) but this was not explored in depth in this study. In this study, a couple who had TB was willing to visit another clinic to get their medicine despites being stigmatized by their community.

There was also suggestion that discrimination of TB patients persisted even after cure, which could be due to inadequate knowledge on the curability of TB. The persistence of stigma after the completion of treatment observed in this study was also described among South Indian patients (30). This might explain the reason why some people did not want to marry those persons who had TB. Discrimination may persist because some TB patients did not recover fully to their pre-illness physical state and, thus, considered permanently weakened or deformed. For unmarried patients in Sabah, TB may affect their prospect of marriage similar to findings in Pakistan (31) and Zambia (10). In a study in Pakistan, stigma was perpetuated by health staff who recommended ‘voluntary social isolation’, to cover their mouth when coughing and to use separate eating-utensils (31). In Kenya, stigma was perpetuated by health staff by isolating TB patients and the use of special measures, such as doormats being impregnated with chemical and the use of gloves before entering TB ward (28). Whether certain advice or activities by healthcare provider contributed towards stigmatization of TB patients was not explored in the present study.

This study contributed considerably to the understanding of many aspects of TB, particularly the knowledge and healthcare-seeking behaviour among TB patients and the community in Sabah. It documented first-hand information on own experiences of patients and from other members of the community. It also covered the opinions expressed by different ethnic groups in seven districts. The findings could not be generalized to the whole population of Sabah in view of its multi-ethnic composition.

Assessing the internal validity of the findings was difficult as the truth is often subjective. There was some bias because the interviewer was health personnel. Perhaps patients may not reveal their true healthcare-seeking behaviour for various reasons despite the assurance of confidentiality and anonymity. However, information from spouses, relatives and the community helped improve the validity of the findings. The findings were considered reliable as all interviews used the same set of questions and, where feedbacks and discussions were concerned, a predetermined guideline was used throughout.

Future research can explore healthcare-seeking behaviours among other ethnic groups and also among immigrant population. In addition, in-depth analysis of stigma would assist in reducing its effect on healthcare-seeking behaviour and on the lives of TB patients.

Although the WHO has provided very generic means in reducing the incidence of TB, such as to increase detection of cases and ensure high cure rate, lack of understanding on the community perspectives, cultural practices and attitudes towards TB may be a reason why TB remains a threat. The findings of this study suggest that there is a need to understand the reasons behind the misconceptions about TB and address the lack of knowledge on TB. Patients need to recognize the symptoms of TB early so that treatment can be initiated promptly. A re-inforcement of the message—“cough of two weeks or more—think TB unless proven otherwise”—need to be conveyed to the community. The curability of the disease must also remain the key point. The messages should be ‘sold’ as a package since fragmentary information will allow the public to create their own understanding to replace the missing pieces. This information package will then have to be channelled through various and appropriate means to cater to the different needs of the population. More support from various groups, such as non-governmental organizations, private companies, and government agencies, need to be garnered to help those affected by TB, especially those who are unemployed or self-employed. By taking such an action, facts about TB will be made open so that the stigma surrounding it will soon fade.

## ACKNOWLEDGEMENTS

The author thanks the Ministry of Health, Malaysia, particularly the officers and health staff of the Sabah Health Department, for their assistance during this study and also all the study participants for their invaluable contribution.
